# Supplementary material for: Bryophytes are predicted to lag behind future climate change despite their high dispersal capacities
Source: Nat Commun. 2020 Nov 5;11:5601. doi: 10.1038/s41467-020-19410-8 (PMC7645420; doi:10.1038/s41467-020-19410-8)
Supplement: Supplementary file 2 — Supplementary Information [file 41467_2020_19410_MOESM2_ESM.pdf]

Supplementary Information

Bryophytes are predicted to lag behind future climate change despite their high dispersal capacities

Zanatta F., Engler R., Collart F., Broennimann O., Mateo R.G., Papp B., Muñoz J., Baurain D., Guisan A. & Vanderpoorten A.

The following supplementary Information is available for this article:

Supplementary Table 1. Average TSS and AUC over 10 replicates of species distribution models (GLM and GBM) for 40 selected bryophyte species in Europe, computed for hold-out data (test set) and the entire dataset (thus using the same data to calibrate and assess the model).

Supplementary Table 2a. Species dispersal traits (spore diameter d, settling velocity Vset, release height Z0), percentage of area gained (number of pixels that become available/number of pixels currently suitable) and lost (number of pixels that become unsuitable/number of pixels currently suitable) in 2050 under the representative concentration pathways (RCP) 4.5 and 8.5 for the Global Circulation Model MPI-ESM-LR. E/C is the ratio, averaged over 30 MigClim replicates, between the rate of range loss and the percentage of newly suitable pixels effectively colonized at the end of the simulation in 2050 when Z0 is assigned to species habitat preferences, windspeed set to maximum, and the probability of long-distance dispersal set to 0 and 0.1, respectively. D is the number of years that is necessary for all pixels that become newly suitable by 2050 to be colonized, for the same two dispersal kernels as for the E/C ratio.

Supplementary Table 2b. percentage of area gained (number of pixels that become available/number of pixels currently suitable) and lost (number of pixels that become unsuitable/number of pixels currently suitable) in 2050 under the representative concentration pathways (RCP) 4.5 and 8.5 for the Had-Gem2-Es Global Circulation Model. E/C is the ratio, averaged over 30 MigClim replicates, between the rate of range loss and the percentage of newly suitable pixels effectively colonized at the end of the simulation in 2050 when Z0 is assigned to species habitat preferences, windspeed set to maximum, and the probability of long-distance dispersal set to 0 and 0.1, respectively. D is the number of years that is necessary for all pixels that become newly suitable by 2050 to be colonized, for the same two dispersal kernels as for the E/C ratio.

Supplementary Figure 1. Distribution of 1 km2 pixel predicted to become climatically suitable and unsuitable in 2050 in European bryophytes of four main biogeographic elements by ensemble climatic niche models under scenario RCP4.5 using the MPI-ESM-LR Global Circulation Model. Colours represent the proportion of species, computed over 10 species per element (see database available from Figshare, DOI: 10.6084/m9.figshare.8289698), for which a pixel becomes suitable (blue) and unsuitable (red). Numbers indicate the average (±S.D.) percentage of the predicted increase (number of pixels that become suitable in 2050) and loss (number of pixels that become unsuitable in 2050), respectively, of suitable area in 2050 as compared to the extent number of suitable pixels.

Supplementary Figure 2. Distribution of 1 km^2 pixel predicted to become climatically suitable and unsuitable in 2050 in European bryophytes of four main biogeographic elements by ensemble climatic niche models under scenario RCP4.5 using the HadGem2-ES Global Circulation Model. Colours represent the proportion of species, computed over 10 species per element (see database available from Figshare, DOI: 10.6084/m9.figshare.8289698), for which a pixel becomes suitable (blue) and unsuitable (red). Numbers indicate the average (±S.D.) percentage of the predicted increase (number of pixels that become suitable in 2050) and loss (number of pixels that become unsuitable in 2050), respectively, of suitable area in 2050 as compared to the extent number of suitable pixels.

Supplementary Figure 3. Distribution of 1 km^2 pixel predicted to become climatically suitable and unsuitable in 2050 in European bryophytes of four main biogeographic elements by ensemble climatic niche models under scenario RCP8.5 using the HadGem2-ES Global Circulation Model. Colours represent the proportion of species, computed over 10 species per element (see database available from Figshare, DOI: 10.6084/m9.figshare.8289698), for which a pixel becomes suitable (blue) and unsuitable (red). Numbers indicate the average (±S.D.) percentage of the predicted increase (number of pixels that become suitable in 2050) and loss (number of pixels that become unsuitable in 2050), respectively, of suitable area in 2050 as compared to the extent number of suitable pixels.

Supplementary Figure 4. Box-plots (showing the 1st and 3d quartiles (upper and lower bounds), 2nd quartile (centre), 1.5* interquartile range (whiskers) and minima-maxima beyond the whiskers) of simulated colonization rates expressed as the ratio (*100), averaged over 30 replicates, between the number of effective colonization events (including effective colonization events that eventually got extinct at the end of the simulation) and the total number of pixels becoming suitable by 2050 in 40 selected bryophyte species in Europe as a function of spore size (a: <20µm; b: 20-50 µm; c: >50 µm), release height Z0, wind speed, and probability of long-distance dispersal P(LDD), with the MPI-ESM-LR Global Circulation Model under climate change scenario RCP4.5.

Supplementary Figure 5. Box-plots (showing the 1st and 3d quartiles (upper and lower bounds), 2nd quartile (centre), 1.5* interquartile range (whiskers) and minima-maxima beyond the whiskers) of simulated colonization rates expressed as the ratio (*100), averaged over 30 replicates, between the number of effective colonization events (including effective colonization events that eventually got extinct at the end of the simulation) and the total number of pixels becoming suitable by 2050 in 40 selected bryophyte species in Europe as a function of spore size (a: <20µm; b: 20-50 µm; c: >50 µm), release height Z0, wind speed, and probability of long-distance dispersal P(LDD), with the HadGem2-ES Global Circulation Model under climate change scenario RCP4.5.

Supplementary Figure 6. Box-plots (showing the 1st and 3d quartiles (upper and lower bounds), 2nd quartile (centre), 1.5* interquartile range (whiskers) and minima-maxima beyond the whiskers) of simulated colonization rates expressed as the ratio (*100), averaged over 30 replicates, between the number of effective colonization events (including effective colonization events that eventually got extinct at the end of the simulation) and the total number of pixels becoming suitable by 2050 in 40 selected bryophyte species in Europe as a function of spore size (a: <20µm; b: 20-50 µm; c: >50 µm), release height Z0, wind speed, and probability of long-distance dispersal P(LDD), with the HadGem2-ES Global Circulation Model under climate change scenario RCP8.5.

Supplementary Table 1. Average TSS and AUC over 10 replicates of species distribution models (GLM and GBM) for 40 selected bryophyte species in Europe, computed for hold-out data (test set) and the entire dataset (thus using the same data to calibrate and assess the model)

Species name	TSS (test set)		AUC (test set)		TSS (entire dataset)		AUC (entire dataset)	
	GLM	GBM	GLM	GBM	GLM	GBM	GLM	GBM
**Artico-Alpine**								
Amphidium lapponicum (Hedw.) Schimp.	0.62	0.64	0.86	0.88	0.62	0.72	0.86	0.93
Anthelia julacea (L.) Dum.	0.84	0.84	0.96	0.97	0.83	0.88	0.96	0.98
Arctoa fulvella (Dicks.) Bruch & Schimp.	0.84	0.78	0.95	0.93	0.82	0.85	0.95	0.98
Cynodontium suecicum (Arnell & C.E.O. Jensen) I. Hagen	0.80	0.82	0.93	0.96	0.79	0.92	0.94	0.98
Cyrtomnium hymenophyllum (Bruch & Schimp.) Holmen	0.90	0.83	0.97	0.93	0.88	0.92	0.97	0.99
Diplophyllum taxifolium (Wahlenb.) Dumort.	0.68	0.69	0.88	0.90	0.68	0.75	0.89	0.94
Grimmia mollis Bruch & Schimp.	0.89	0.75	0.96	0.90	0.85	0.92	0.96	0.98

Gymnomitrion corallioides Nees	0.80	0.81	0.93	0.93	0.81	0.88	0.94	0.97
Paludella squarrosa (Hedw.) Brid.	0.58	0.68	0.85	0.90	0.58	0.71	0.85	0.92
Sphagnum aongstroemii C. Hartm.	0.80	0.86	0.93	0.97	0.80	0.90	0.94	0.98
Atlantic	**Atlantic**							
Anastrophyllum donnianum (Hook.) Steph.	0.99	0.97	1.00	1.00	0.98	0.99	1.00	1.00
Bazzania pearsonii Steph.	0.96	0.96	0.98	0.99	0.96	1.00	1.00	1.00
Dicranum scottianum Turner	0.89	0.90	0.98	0.98	0.90	0.93	0.98	0.99
Herbertus stramineus (Dumort.) Trevis.	0.95	0.95	0.99	0.99	0.95	0.97	0.99	0.99
Glyphomitrium daviesii (Dicks.) Brid.	0.98	0.97	1.00	0.99	0.98	0.99	1.00	0.99
Mastigophora woodsii (Hook.) Nees	0.99	0.95	1.00	0.99	0.99	0.98	1.00	1.00
Myurium hochstetteri (Schimp.) Kindb.	0.96	0.96	0.98	0.98	1.00	1.00	1.00	1.00
Ptychomitrium polyphyllum	0.83	0.84	0.97	0.97	0.83	0.87	0.97	0.98

(Sw.) Bruch & Schimp.								
Saccogyna viticulosa (L.) Dumort.	0.92	0.92	0.98	0.98	0.92	0.95	0.98	0.99
Ulota calvescens Carrington	0.88	0.90	0.97	0.97	0.88	0.90	0.97	0.98
Mediterranean	**Mediterranean**							
Bartramia stricta Brid.	0.75	0.73	0.91	0.90	0.74	0.89	0.91	0.98
Corsinia coriandrina (Spreng.) Lindb.	0.81	0.80	0.94	0.94	0.80	0.96	0.94	0.99
Fabronia pusilla Raddi	0.71	0.77	0.88	0.93	0.71	0.93	0.90	0.99
Fossombronia caespitiformis (Raddi) De Not. ex Rabenh.	0.76	0.77	0.93	0.92	0.77	0.90	0.94	0.98
Habrodon perpusillus (De Not.) Lindb.	0.71	0.75	0.90	0.92	0.69	0.87	0.91	0.98
Homalothecium aureum (Spruce) H. Rob.	0.82	0.84	0.94	0.95	0.82	0.93	0.94	0.99
Leptodon smithii (Dicks. ex Hedw.) F. Weber & D. Mohr	0.74	0.76	0.92	0.93	0.72	0.80	0.92	0.96
Oxymitra incrassata	0.70	0.78	0.88	0.91	0.63	0.93	0.89	1.00

(Brot.) Sérgio & Sim-Sim								
Scleropodium touretii (Brid.) L.F. Koch	0.67	0.73	0.91	0.93	0.66	0.76	0.92	0.96
Scorpiurium circinatum (Brid.) M. Fleisch. & Loeske	0.77	0.78	0.92	0.94	0.75	0.83	0.93	0.97
Wide Temperate	**Wide temperate**							
Amphidium mougeotii (Bruch & Schimp.) Schimp.	0.60	0.66	0.88	0.90	0.60	0.68	0.88	0.91
Anomodon viticulosus (Hedw.) Hook. & Taylor	0.63	0.65	0.86	0.88	0.63	0.67	0.86	0.89
Atrichum undulatum (Hedw.) P. Beauv.	0.67	0.67	0.88	0.89	0.66	0.68	0.88	0.89
Diplophyllum albicans (L.) Dumort.	0.70	0.73	0.90	0.92	0.70	0.74	0.90	0.93
Frullania dilata (L.) Dumort.	0.66	0.68	0.88	0.90	0.65	0.68	0.88	0.90
Leucodon sciuroides (Hedw.) Schwägr.	0.58	0.59	0.84	0.86	0.57	0.60	0.84	0.87

Metzgeria furcata (L.) Corda	0.66	0.66	0.88	0.89	0.65	0.66	0.88	0.89
Orthotrichum affine Brid.	0.69	0.69	0.89	0.90	0.69	0.70	0.89	0.91
Orthotrichum lyellii Hook. & Taylor	0.69	0.70	0.90	0.91	0.69	0.71	0.90	0.92
Plagiomnium undulatum (Hedw.) T.J. Kop.	0.68	0.69	0.88	0.89	0.67	0.70	0.88	0.90

Supplementary Table 2a. Species dispersal traits (spore diameter d, settling velocity Vset, release height Z0), percentage of area gained (number of pixels that become available/number of pixels currently suitable) and lost (number of pixels that become unsuitable/number of pixels currently suitable) in 2050 under the representative concentration pathways (RCP) 4.5 and 8.5 for the Global Circulation Model MPI-ESM-LR. E/C is the ratio, averaged over 30 MigClim replicates, between the rate of range loss and the percentage of newly suitable pixels effectively colonized at the end of the simulation in 2050 when Z0 is assigned to species habitat preferences, windspeed set to maximum, and the probability of long-distance dispersal set to 0 and 0.1, respectively. D is the number of years that is necessary for all pixels that become newly suitable by 2050 to be colonized, for the same two dispersal kernels as for the E/C ratio.

Species name	d (µm)	V_{set} (m/s)	Z0 (m)	MPI 4.5						MPI 8.5					
				Lost habitat (%)	New habitat (%)	E/C; Ldd=0	E/C; Ldd=0.1	D, Ldd=0	D, Ldd=0.1	Lost habitat (%)	New habitat (%)	E/C; Ldd=0	E/C; Ldd=0.1	D, Ldd=0	D, Ldd=0.1
**Artic-Alpine**				40 ± 13	7 ± 8	104.19 ± 184.25	77.27 ± 109.73			42 ± 14	9 ± 8	145.01 ± 313.11	95.14 ± 164.47		
Amphidium lapponicum (Hedw.) Schimp.	10	$3.36\ 10^{-3}$	1	40	3	37.17	37.16	0	0	41	3	40.93	40.91	> 500	> 500
Anthelia julacea (L.) Dum.	14	$6.59\ 10^{-3}$	0.03	22	12	27.83	24.26	> 500	> 500	23	13	27.57	24.08	> 500	> 500
Arctoa fulvella (Dicks.) Bruch & Schimp.	23	$1.78\ 10^{-2}$	1	18	11	18.01	17.35	0	0	21	11	21.91	21.41	> 500	20
Cynodontium suecicum (Arnell & C.E.O. Jensen) I. Hagen	22.5	$1.70\ 10^{-2}$	1	45	27	55.53	53.54	> 500	> 500	45	30	48.47	44.66	109	0

Species name	d (µm)	V_{set} (m/s)	Z0 (m)	MPI 4.5						MPI 8.5					
				Lost habitat (%)	New habitat (%)	E/C; Ldd=0	E/C; Ldd=0.1	D, Ldd=0	D, Ldd=0.1	Lost habitat (%)	New habitat (%)	E/C; Ldd=0	E/C; Ldd=0.1	D, Ldd=0	D, Ldd=0.1
Cyrtomnium hymenophyllum (Bruch & Schimp.) Holmen	150	7.57 10^{-1}	1	43	1	626.78	386.37	> 500	> 500	6	0	1035.05	561.15	> 500	> 500
Diplophyllum taxifolium (Wahlenb.) Dumort.	14	6.59 10^{-3}	0.03	38	6	54.80	53.28	> 500	> 500	39	7	49.27	41.32	> 500	65
Grimmia mollis Bruch & Schimp.	11.5	4.45 10^{-3}	1	57	9	56.28	56.14	32	0	30	8	71.33	70.86	> 500	> 500
Gymnomitrion corallioides Nees	14	6.59 10^{-3}	1	30	5	40.94	28.15	> 500	0	0	5	35.76	31.83	> 500	> 500
Paludella squarrosa (Hedw.) Brid.	17.5	1.03 10^{-2}	0.03	50	3	56.63	51.78	257	26	12	4	56.25	54.47	> 500	76
Sphagnum aongstroemii C. Hartm.	22.5	1.70 10^{-2}	0.03	55	11	67.93	64.69	> 500	> 500	16	0	63.53	60.75	> 500	> 500
**Atlantic**				22 ± 9	24 ± 10	77.19 ± 90.33	56.41 ± 68.20			31 ± 12	26 ± 11	179.24 ± 269.72	125.15 ± 202.43		

Species name	d (µm)	V_{set} (m/s)	Z0 (m)	MPI 4.5						MPI 8.5					
				Lost habitat (%)	New habitat (%)	E/C; Ldd=0	E/C; Ldd=0.1	D, Ldd=0	D, Ldd=0.1	Lost habitat (%)	New habitat (%)	E/C; Ldd=0	E/C; Ldd=0.1	D, Ldd=0	D, Ldd=0.1
Anastrophyllum donnianum (Hook.) Steph.	150	$7.57\ 10^{-1}$	1	29	37	118.79	61.88	> 500	> 500	41	48	197.16	96.66	> 500	> 500
Bazzania pearsonii Steph.	150	$7.57\ 10^{-1}$	0.03	34	13	308.35	243.13	> 500	> 500	52	17	850.03	665.80	> 500	> 500
Dicranum scottianum Turner	27.5	$2.54\ 10^{-2}$	1	25	16	29.41	27.36	> 500	> 500	27	17	33.13	27.47	> 500	0
Herbertus stramineus (Dumort.) Trevis.	150	$7.57\ 10^{-1}$	1	12	26	30.52	26.46	> 500	> 500	16	30	40.63	32.37	> 500	> 500
Glyphomitrium daviesii (Dicks.) Brid.	45	$6.81\ 10^{-2}$	1	26	37	56.48	37.19	> 500	> 500	32	42	82.56	47.78	> 500	96
Mastigophora woodsii (Hook.) Nees	150	$7.57\ 10^{-1}$	0.03	10	18	125.89	73.00	> 500	> 500	19	24	446.41	252.83	> 500	> 500
Myurium hochstetteri (Schimp.) Kindb.	150	$7.57\ 10^{-1}$	1	21	41	30.21	28.36	> 500	> 500	28	31	39.71	37.55	> 500	> 500
Ptychomitrium polyphyllum	12	$4.84\ 10^{-3}$	1	35	14	33.69	31.00	0	0	45	14	49.67	45.69	> 500	15

Species name	d (µm)	V_{set} (m/s)	Z0 (m)	MPI 4.5						MPI 8.5					
				Lost habitat (%)	New habitat (%)	E/C; Ldd=0	E/C; Ldd=0.1	D, Ldd=0	D, Ldd=0.1	Lost habitat (%)	New habitat (%)	E/C; Ldd=0	E/C; Ldd=0.1	D, Ldd=0	D, Ldd=0.1
(Sw.) Bruch & Schimp.															
Saccogyna viticulosa (L.) Dumort.	13.5	$6.13\ 10^{-3}$	1	9	13	9.44	8.41	44	0	11	15	14.00	11.25	> 500	6
Ulota calvescens Carrington	24	$1.94\ 10^{-2}$	10	27	21	29.09	27.31	> 500	> 500	34	22	39.11	34.10	> 500	35
**Mediterranean**				32 ± 10	38 ± 14	331.24 ± 607.72	61.01 ± 54.65			35 ± 10	39 ± 15	392.67 ± 702.46	59.90 ± 54.54		
Bartramia stricta Brid.	29	$2.83\ 10^{-2}$	1	27	34	31.51	29.33	> 500	> 500	29	34	32.32	29.47	> 500	20
Corsinia coriandrina (Spreng.) Lindb.	120	$4.84\ 10^{-1}$	0.03	42	50	1022.65	187.27	> 500	> 500	48	54	1498.36	196.77	> 500	357
Fabronia pusilla Raddi	11.5	$4.45\ 10^{-3}$	10	41	31	47.66	45.15	> 500	> 500	43	31	46.72	42.81	> 500	0
Fossombronia caespitiformis (Raddi) De Not. ex Rabenh.	47	$7.43\ 10^{-2}$	0.03	25	38	115.11	42.56	> 500	> 500	25	38	118.37	37.40	> 500	110

Species name	d (µm)	V_{set} (m/s)	Z0 (m)	MPI 4.5						MPI 8.5					
				Lost habitat (%)	New habitat (%)	E/C; Ldd=0	E/C; Ldd=0.1	D, Ldd=0	D, Ldd=0.1	Lost habitat (%)	New habitat (%)	E/C; Ldd=0	E/C; Ldd=0.1	D, Ldd=0	D, Ldd=0.1
Habrodon perpusillus (De Not.) Lindb.	20	1.35 10^{-2}	10	29	18	29.25	28.27	> 500	0	33	17	34.45	32.76	> 500	1
Homalothecium aureum (Spruce) H. Rob.	16.5	9.16 10^{-3}	0.03	53	45	151.81	72.56	> 500	> 500	57	49	175.75	59.51	> 500	38
Leptodon smithii (Dicks. ex Hedw.) F. Weber & D. Mohr	16	8.61 10^{-3}	10	25	39	27.35	25.43	> 500	> 500	31	37	32.93	31.33	> 500	> 500
Oxymitra incrassata (Brot.) Sérgio & Sim-Sim	137.5	6.36 10^{-1}	0.03	28	71	1825.42	127.41	> 500	> 500	28	74	1919.68	112.41	> 500	> 500
Scleropodium touretii (Brid.) L.F. Koch	16	8.61 10^{-3}	0.03	21	20	26.54	22.50	> 500	14	25	20	32.35	26.99	> 500	121
Scorpiurium circinatum (Brid.) M. Fleisch. & Loeske	16	8.61 10^{-3}	1	26	39	35.11	29.63	> 500	> 500	29	40	35.80	29.53	> 500	0
**Wide temperate**				32 ± 5	15 ± 2	45.26 ± 18.56	33.62 ± 7.06			37 ± 4	16 ± 3	54.38 ± 19.20	38.97 ± 5.82		

Species name	d (µm)	V_{set} (m/s)	Z0 (m)	MPI 4.5						MPI 8.5					
				Lost habitat (%)	New habitat (%)	E/C; Ldd=0	E/C; Ldd=0.1	D, Ldd=0	D, Ldd=0.1	Lost habitat (%)	New habitat (%)	E/C; Ldd=0	E/C; Ldd=0.1	D, Ldd=0	D, Ldd=0.1
Amphidium mougeotii (Bruch & Schimp.) Schimp.	11	4.07 10^{-3}	1	42	18	42.96	42.38	> 500	> 500	44	20	44.31	43.66	> 500	0
Anomodon viticulosus (Hedw.) Hook. & Taylor	16	8.61 10^{-3}	1	28	15	36.90	30.28	> 500	> 500	35	16	48.03	35.41	> 500	0
Atrichum undulatum (Hedw.) P. Beauv.	18	1.09 10^{-2}	0.03	39	16	72.73	43.51	> 500	> 500	41	16	82.20	46.51	> 500	34
Diplophyllum albicans (L.) Dumort.	13	5.68 10^{-3}	0.03	34	19	65.79	36.42	> 500	24	40	19	76.83	42.88	> 500	21
Frullania dilata (L.) Dumort.	50	8.41 10^{-2}	10	23	14	26.43	23.68	> 500	> 500	30	14	36.81	29.83	> 500	0
Leucodon sciuroides (Hedw.) Schwägr.	23	1.78 10^{-2}	10	27	18	35.97	29.51	> 500	> 500	34	20	43.45	33.98	> 500	0
Metzgeria furcata (L.) Corda	26,5	2.36 10^{-2}	10	28	16	34.81	29.63	> 500	> 500	34	17	42.38	33.99	> 500	0

Species name	d (µm)	V_{set} (m/s)	Z0 (m)	MPI 4.5						MPI 8.5					
				Lost habitat (%)	New habitat (%)	E/C; Ldd=0	E/C; Ldd=0.1	D, Ldd=0	D, Ldd=0.1	Lost habitat (%)	New habitat (%)	E/C; Ldd=0	E/C; Ldd=0.1	D, Ldd=0	D, Ldd=0.1
Orthotrichum affine Brid.	20	$1.35\ 10^{-2}$	10	35	11	35.17	32.93	2	0	41	12	44.85	41.14	> 500	0
Orthotrichum lyellii Hook. & Taylor	33	$3.66\ 10^{-2}$	10	28	11	27.12	26.08	0	0	36	12	38.96	36.12	> 500	> 500
Plagiomnium undulatum (Hedw.) T.J. Kop.	28	$2.64\ 10^{-2}$	0.03	33	16	74.73	41.77	> 500	> 500	37	17	85.96	46.20	> 500	46
**All species**															
Average ± SD				31.5 ± 11.4	21.5 ± 14.9	139.5 ± 328.7	57.1 ± 69.3			36.2 ± 11.9	22.8 ± 15.5	192.8 ± 411.3	79.8 ± 132.3		
Median				28.5	17.0	39.0	34.6			34.5	17.0	45.8	41.0		

Supplementary Table 2b. percentage of area gained (number of pixels that become available/number of pixels currently suitable) and lost (number of pixels that become unsuitable/number of pixels currently suitable) in 2050 under the representative concentration pathways (RCP) 4.5 and 8.5 for the Had-Gem2-Es Global Circulation Model. E/C is the ratio, averaged over 30 MigClim replicates, between the rate of range loss and the percentage of newly suitable pixels effectively colonized at the end of the simulation in 2050 when Z0 is assigned to species habitat preferences, windspeed set to maximum, and the probability of long-distance dispersal set to 0 and 0.1, respectively. D is the number of years that is necessary for all pixels that become newly suitable by 2050 to be colonized, for the same two dispersal kernels as for the E/C ratio.

Species name	HE 4.5						HE 8.5					
	Lost habitat (%)	New habitat (%)	E/C; Ldd=0	E/C; Ldd=0.1	D, Ldd=0	D, Ldd=0.1	Lost habitat (%)	New habitat (%)	E/C; Ldd=0	E/C; Ldd=0.1	D, Ldd=0	D, Ldd=0.1
**Artico-Alpine**	55.18 ± 14.04	7.70 ± 9.98	554.67 ± 1559.99	228.56 ± 545.87			62.89 ± 15.30	7.33 ± 9.53	782.33 ± 2247.00	258.96 ± 617.09		
Amphidium lapponicum (Hedw.) Schimp.	53.36	1.16	53.62	53.60	> 500	> 500	59.28	1.14	59.52	59.50	> 500	> 500
Anthelia julacea (L.) Dum.	34.95	11.46	46.51	36.25	> 500	> 500	40.87	10.46	55.34	42.27	> 500	> 500
Arctoa fulvella (Dicks.) Bruch & Schimp.	33.76	0.90	33.92	33.79	> 500	14	40.21	0.72	40.13	40.00	0	0
Cynodontium suecicum (Arnell & C.E.O. Jensen) I. Hagen	54.44	32.75	58.38	54.36	> 500	0	61.36	30.55	64.74	61.31	> 500	1
Cyrtomnium hymenophyllum (Bruch & Schimp.) Holmen	66.63	2.38	4994.20	1781.48	> 500	> 500	77.62	1.81	7177.11	2014.54	> 500	> 500
Diplophyllum taxifolium (Wahlenb.) Dumort.	53.26	7.47	75.25	55.38	> 500	62	59.79	8.13	91.60	62.43	> 500	52
Grimmia mollis Bruch & Schimp.	71.36	0.91	71.50	71.48	> 500	> 500	80.69	0.09	81.05	81.05	> 500	> 500
Gymnomitrion corallioides Nees	47.01	1.38	48.78	47.12	> 500	211	55.78	1.09	57.14	55.86	> 500	> 500
Paludella squarrosa (Hedw.) Brid.	62.04	4.41	71.21	65.27	> 500	70	69.00	4.49	85.38	75.02	> 500	76
Sphagnum aongstroemii C. Hartm.	74.99	14.13	93.36	86.81	> 500	> 500	84.36	14.83	111.24	97.66	> 500	> 500
**Atlantic**	50.14 ± 14.34	30.98 ± 11.54	571.99 ± 1230.26	321.04 ± 651.68			57.17 ± 14.73	40.23 ± 15.81	1167.32 ± 2664.88	522.75 ± 1128.28		

Species name	HE 4.5						HE 8.5					
	Lost habitat (%)	New habitat (%)	E/C; Ldd=0	E/C; Ldd=0.1	D, Ldd=0	D, Ldd=0.1	Lost habitat (%)	New habitat (%)	E/C; Ldd=0	E/C; Ldd=0.1	D, Ldd=0	D, Ldd=0.1
Anastrophyllum donnianum (Hook.) Steph.	51.81	50.14	224.09	111.49	> 500	> 500	59.11	53.91	279.01	131.21	> 500	> 500
Bazzania pearsonii Steph.	70.73	45.02	3995.72	2129.34	> 500	> 500	79.92	65.92	8552.22	3644.28	> 500	> 500
Dicranum scottianum Turner	46.45	23.25	70.20	46.37	> 500	0	57.31	28.11	102.04	57.30	> 500	1
Herbertus stramineus (Dumort.) Trevis.	24.38	32.00	77.45	54.30	> 500	> 500	29.06	37.72	90.17	57.61	> 500	> 500
Glyphomitrium daviesii (Dicks.) Brid.	53.98	37.84	127.41	66.29	> 500	67	57.49	51.75	187.53	83.80	> 500	57
Mastigophora woodsii (Hook.) Nees	40.58	33.05	904.74	520.99	> 500	> 500	49.78	51.84	2051.81	916.23	> 500	> 500
Myurium hochstetteri (Schimp.) Kindb.	64.67	15.06	146.31	132.81	> 500	> 500	72.94	45.17	213.90	170.95	> 500	> 500
Ptychomitrium polyphyllum (Sw.) Bruch & Schimp.	63.35	17.82	72.59	63.48	> 500	32	65.60	18.70	75.29	65.69	> 500	35
Saccogyna viticulosa (L.) Dumort.	34.22	21.69	46.91	34.20	> 500	0	41.01	22.95	57.43	41.00	> 500	0
Ulota calvescens Carrington	51.19	11.54	54.46	51.17	> 500	0	59.51	26.26	63.76	59.48	> 500	0
**Mediterranean**	49.29 ± 11.36	39.32 ± 19.57	806.84 ± 1593.31	93.67 ± 94.52			56.97 ± 12.17	38.00 ± 17.64	911.88 ± 1725.18	112.64 ± 118.70		
Bartramia stricta Brid.	44.23	33.06	56.12	44.22	> 500	39	50.35	33.36	69.36	50.34	> 500	23
Corsinia coriandrina (Spreng.) Lindb.	63.88	57.89	2393.18	313.89	> 500	402	71.64	55.94	2876.20	402.89	> 500	434

Species name	HE 4.5						HE 8.5					
	Lost habitat (%)	New habitat (%)	E/C; Ldd=0	E/C; Ldd=0.1	D, Ldd=0	D, Ldd=0.1	Lost habitat (%)	New habitat (%)	E/C; Ldd=0	E/C; Ldd=0.1	D, Ldd=0	D, Ldd=0.1
Fabronia pusilla Raddi	61.24	40.01	79.40	61.13	> 500	0	70.74	34.36	123.13	70.60	> 500	0
Fossombronia caespitiformis (Raddi) De Not. ex Rabenh.	36.39	26.76	119.98	46.53	> 500	211	40.01	25.18	126.51	51.46	> 500	335
Habrodon perpusillus (De Not.) Lindb.	46.94	16.91	57.58	46.91	> 500	1	55.72	20.24	72.02	55.63	> 500	0
Homalothecium aureum (Spruce) H. Rob.	68.39	56.65	359.24	72.55	> 500	33	76.67	56.02	495.58	83.96	> 500	29
Leptodon smithii (Dicks. ex Hedw.) F. Weber & D. Mohr	48.95	27.15	51.98	48.91	> 500	2	57.83	27.12	61.73	57.76	> 500	0
Oxymitra incrassata (Brot.) Sérgio & Sim-Sim	44.35	76.86	4844.14	220.29	> 500	374	48.89	72.35	5150.51	249.58	> 500	393
Scleropodium touretii (Brid.) L.F. Koch	36.20	18.83	54.30	39.92	> 500	58	46.55	19.68	80.30	52.85	> 500	59
Scorpiurium circinatum (Brid.) M. Fleisch. & Loeske	42.35	36.06	52.42	42.33	> 500	2	51.30	35.79	63.47	51.30	> 500	9
**Wide temperate**	73.10 ± 5.79	18.25 ± 2.40	98.20 ± 31.54	66.22 ± 7.43			64.12 ± 4.88	15.31 ± 2.83	119.85 ± 43.34	75.05 ± 7.98		
Amphidium mougeotii (Bruch & Schimp.) Schimp.	63.59	20.29	58.11	57.66	> 500	0	57.76	19.28	64.41	63.50	> 500	0
Anomodon viticulosus (Hedw.) Hook. & Taylor	77.72	19.17	99.66	66.05	> 500	1	66.06	15.82	119.55	77.62	> 500	0
Atrichum undulatum (Hedw.) P. Beauv.	78.05	19.12	152.61	75.35	> 500	45	68.60	15.66	191.80	84.84	> 500	52

Species name	HE 4.5						HE 8.5					
	Lost habitat (%)	New habitat (%)	E/C; Ldd=0	E/C; Ldd=0.1	D, Ldd=0	D, Ldd=0.1	Lost habitat (%)	New habitat (%)	E/C; Ldd=0	E/C; Ldd=0.1	D, Ldd=0	D, Ldd=0.1
Diplophyllum albicans (L.) Dumort.	72.51	20.45	104.00	68.44	> 500	18	67.19	17.94	110.22	73.60	> 500	18
Frullania dilata (L.) Dumort.	64.76	17.22	75.60	56.23	> 500	0	56.32	14.01	87.94	64.67	> 500	0
Leucodon sciuroides (Hedw.) Schwägr.	71.94	20.57	89.74	61.96	> 500	0	62.04	17.59	107.80	71.88	> 500	0
Metzgeria furcata (L.) Corda	70.85	18.58	76.39	60.53	> 500	0	60.65	15.79	88.37	70.72	> 500	0
Orthotrichum affine Brid.	82.22	13.17	89.50	71.81	> 500	0	71.90	9.85	124.44	82.12	> 500	0
Orthotrichum lyellii Hook. & Taylor	74.37	15.31	83.55	65.55	> 500	4	65.57	11.84	105.52	74.34	> 500	9
Plagiomnium undulatum (Hedw.) T.J. Kop.	75.03	18.61	152.93	78.59	> 500	71	65.09	15.28	198.48	87.22	> 500	92
**All species**												
Average ± SD	56.9 ± 15.0	23.4 ± 17.0	507.9 ± 1250.8	177.4 ± 424.0			60.3 ± 12.4	25.2 ± 18.9	745.3 ± 1909.1	242.3 ± 645.4		
Median	57.8	19.1	76.9	61.5			59.6	19.5	96.8	70.7		

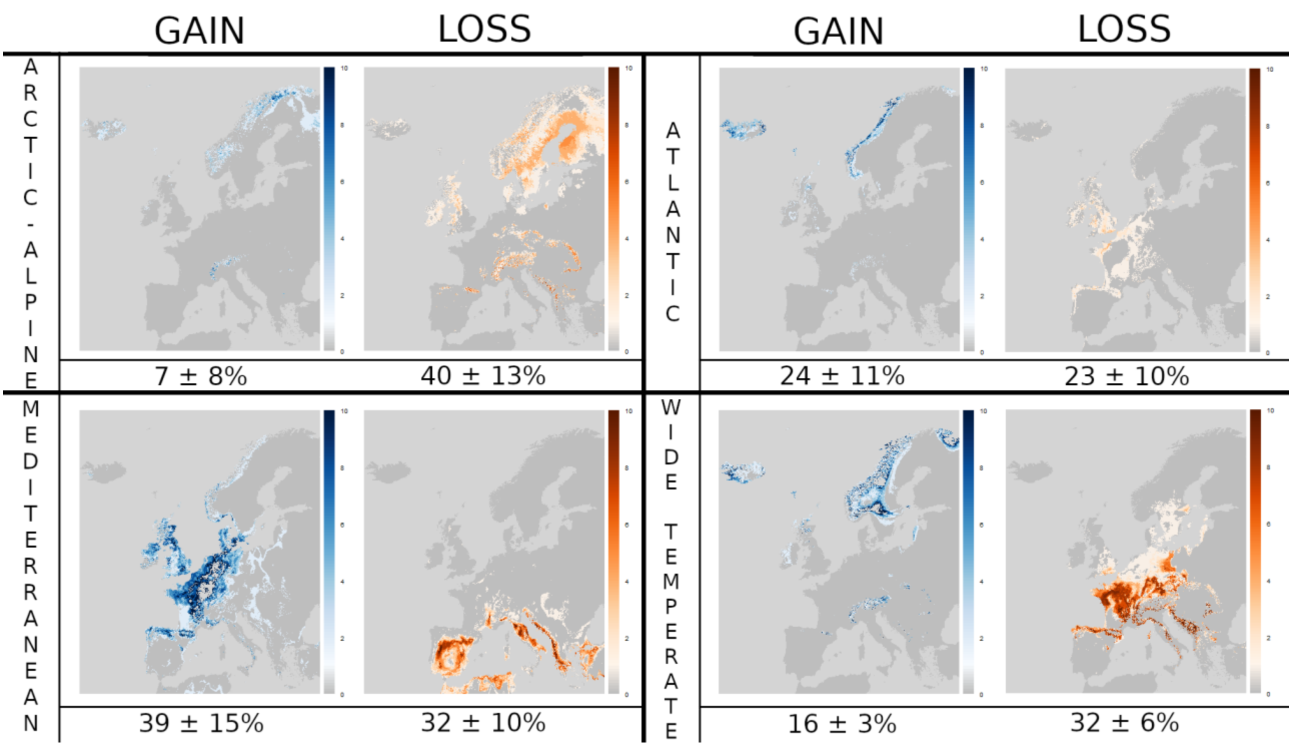

Supplementary Figure 1. Distribution of 1 km^2 pixel predicted to become climatically suitable and unsuitable in 2050 in European bryophytes of four main biogeographic elements by ensemble climatic niche models under scenario RCP4.5 using the MPI-ESM-LR Global Circulation Model. Colours represent the proportion of species, computed over 10 species per element (see database available from Figshare, DOI: 10.6084/m9.figshare.8289698), for which a pixel becomes suitable (blue) and unsuitable (red). Numbers indicate the average (±S.D.) percentage of the predicted increase (number of pixels that become suitable in 2050) and loss (number of pixels that become unsuitable in 2050), respectively, of suitable area in 2050 as compared to the extent number of suitable pixels.

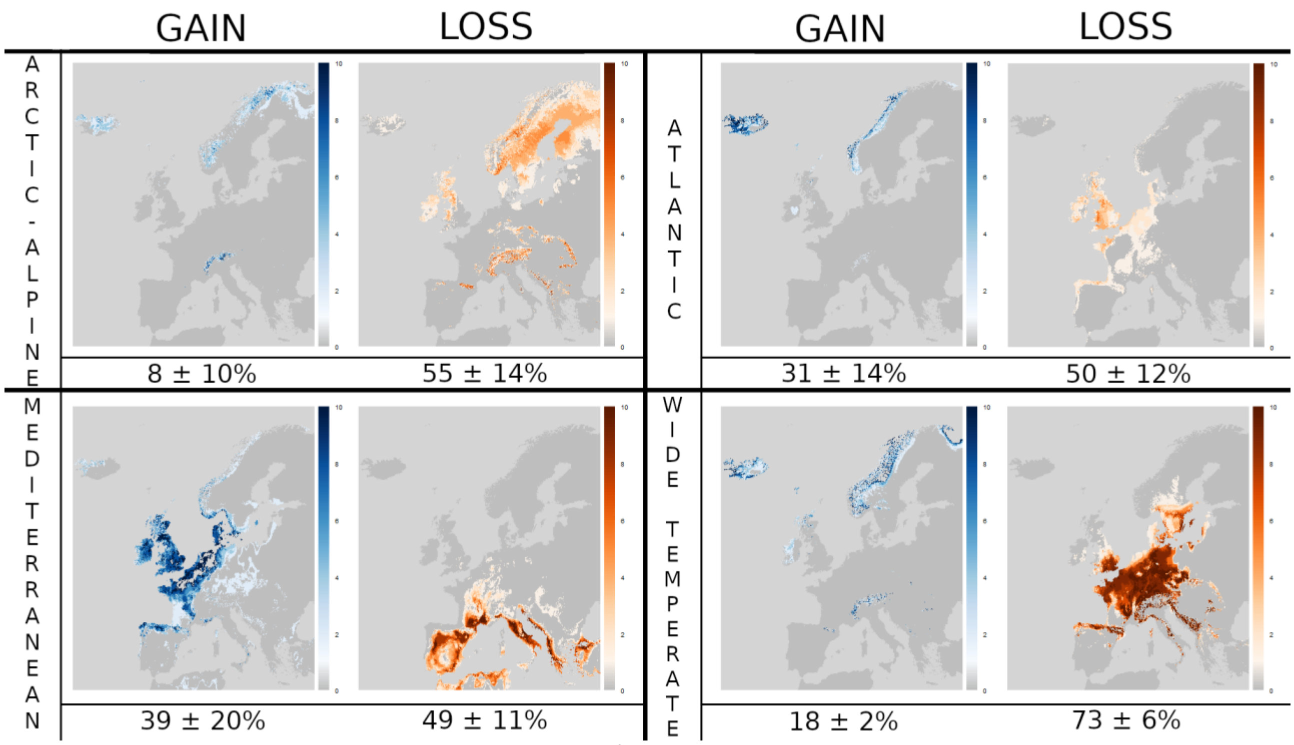

Supplementary Figure 2. Distribution of 1 km^2 pixel predicted to become climatically suitable and unsuitable in 2050 in European bryophytes of four main biogeographic elements by ensemble climatic niche models under scenario RCP4.5 using the HadGem2-ES Global Circulation Model. Colours represent the proportion of species, computed over 10 species per element (see database available from Figshare, DOI: 10.6084/m9.figshare.8289698), for which a pixel becomes suitable (blue) and unsuitable (red). Numbers indicate the average (±S.D.) percentage of the predicted increase (number of pixels that become suitable in 2050) and loss (number of pixels that become unsuitable in 2050), respectively, of suitable area in 2050 as compared to the extent number of suitable pixels.

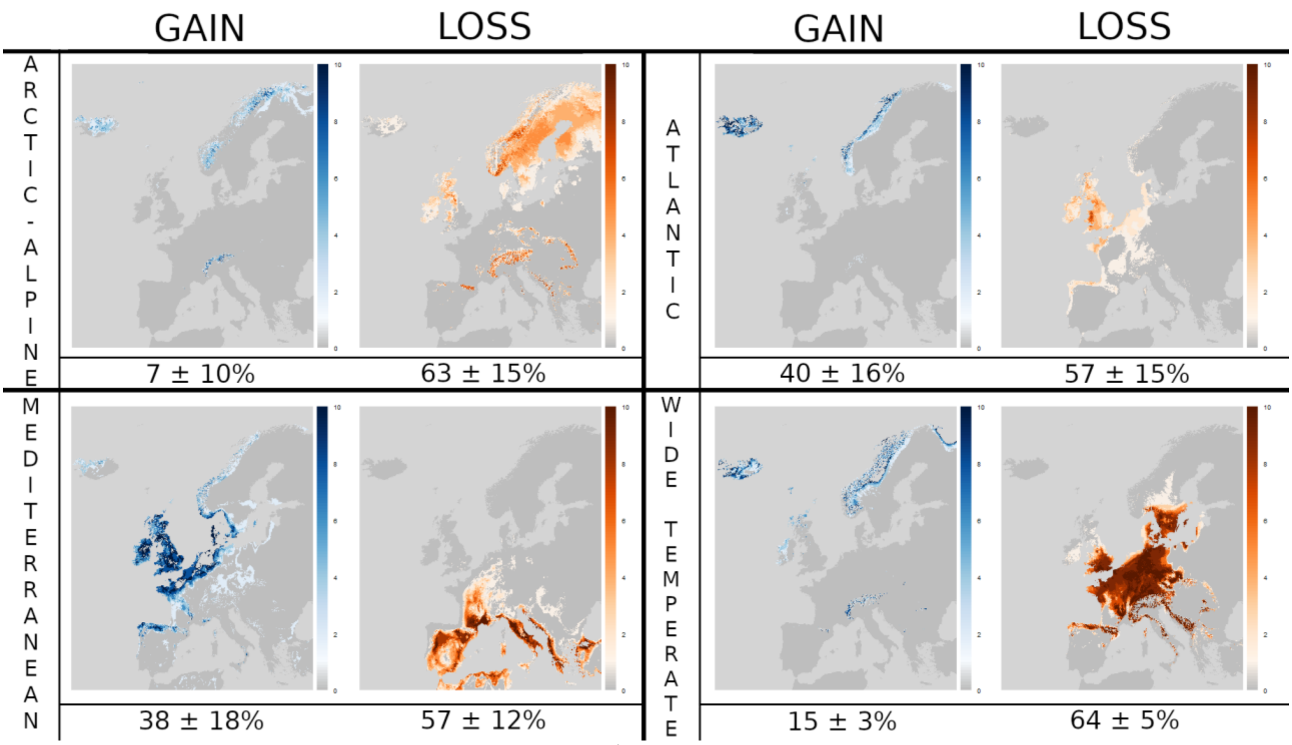

Supplementary Figure 3. Distribution of 1 km^2 pixel predicted to become climatically suitable and unsuitable in 2050 in European bryophytes of four main biogeographic elements by ensemble climatic niche models under scenario RCP8.5 using the HadGem2-ES Global Circulation Model. Colours represent the proportion of species, computed over 10 species per element (see database available from Figshare, DOI: 10.6084/m9.figshare.8289698), for which a pixel becomes suitable (blue) and unsuitable (red). Numbers indicate the average (±S.D.) percentage of the predicted increase (number of pixels that become suitable in 2050) and loss (number of pixels that become unsuitable in 2050), respectively, of suitable area in 2050 as compared to the extent number of suitable pixels.

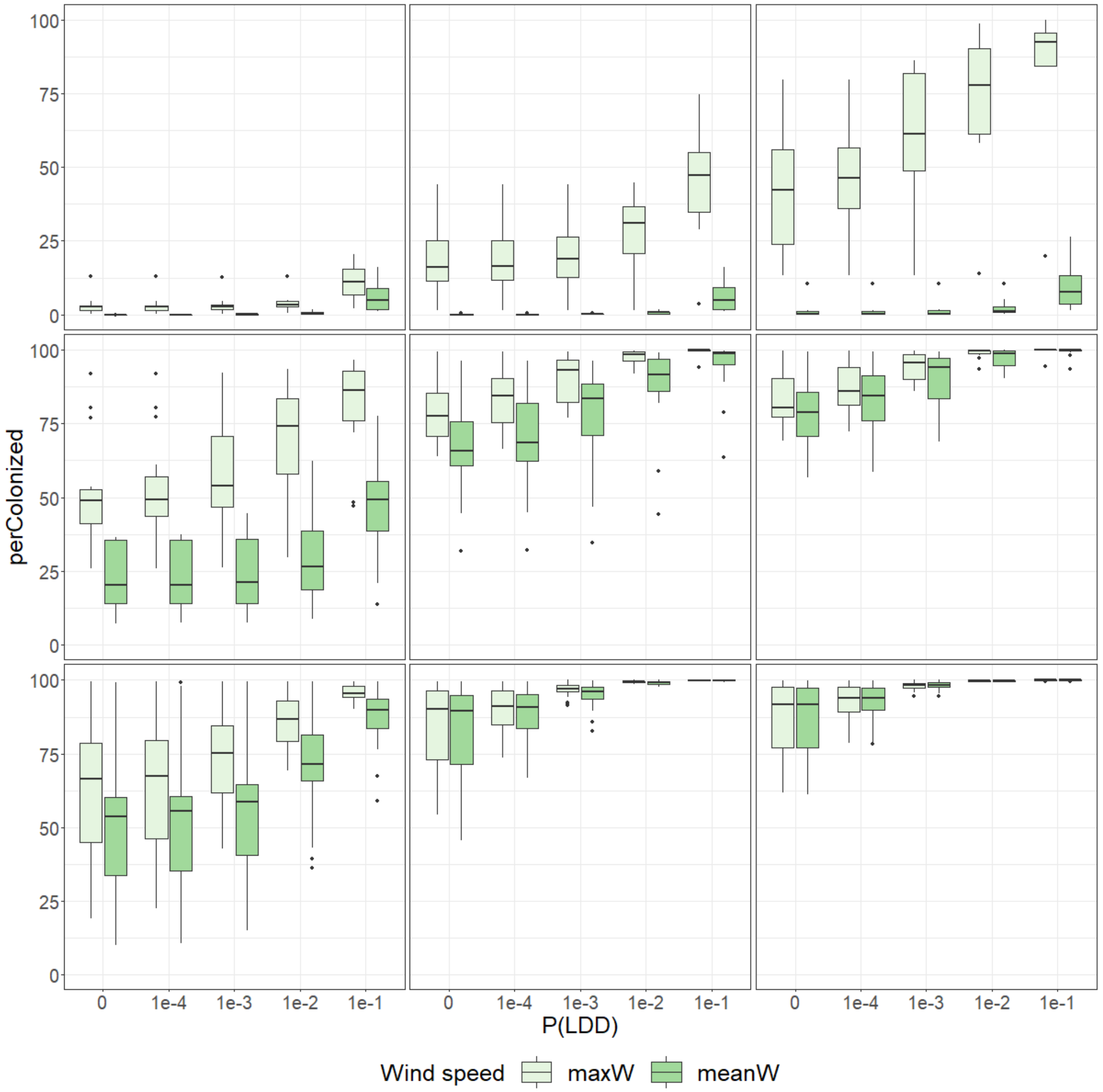

Supplementary Figure 4. Box-plots (showing the 1st and 3^{d} quartiles (upper and lower bounds), 2nd quartile (centre), 1.5* interquartile range (whiskers) and minima-maxima beyond the whiskers) of simulated colonization rates expressed as the ratio (*100), averaged over 30 replicates, between the number of effective colonization events (including effective colonization events that eventually got extinct at the end of the simulation) and the total number of pixels becoming suitable by 2050 in 40 selected bryophyte species in Europe as a function of spore size (a: <20µm; b: 20-50 µm; c: >50 µm), release height Z0, wind speed, and probability of long-distance dispersal P(LDD), with the MPI-ESM-LR Global Circulation Model under climate change scenario RCP4.5.

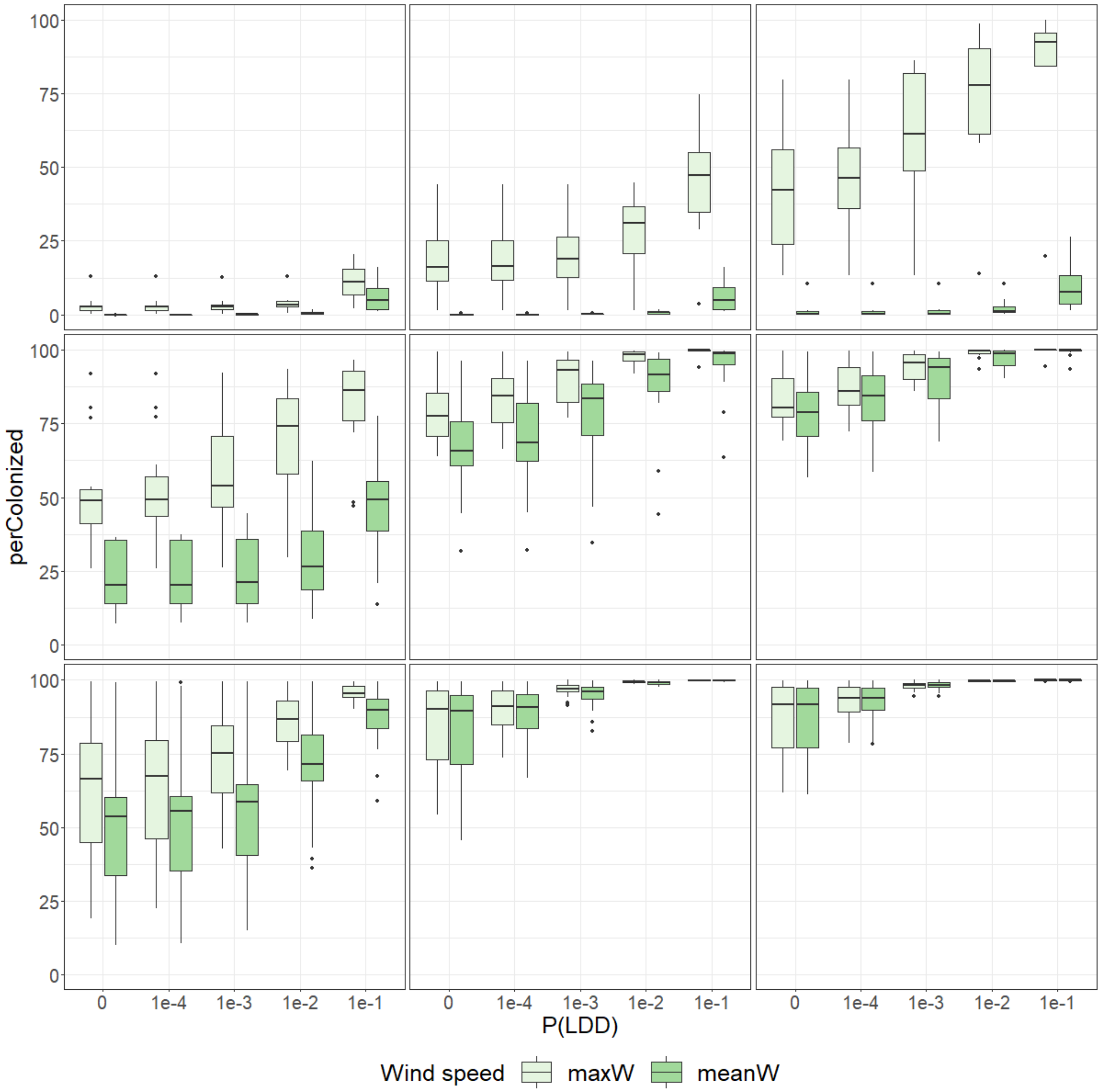

Supplementary Figure 5. Box-plots (showing the 1st and 3d quartiles (upper and lower bounds), 2nd quartile (centre), 1.5* interquartile range (whiskers) and minima-maxima beyond the whiskers) of simulated colonization rates expressed as the ratio (*100), averaged over 30 replicates, between the number of effective colonization events (including effective colonization events that eventually got extinct at the end of the simulation) and the total number of pixels becoming suitable by 2050 in 40 selected bryophyte species in Europe as a function of spore size (a: <20µm; b: 20-50 µm; c: >50 µm), release height Z0, wind speed, and probability of long-distance dispersal P(LDD), with the HadGem2-ES Global Circulation Model under climate change scenario RCP4.5.

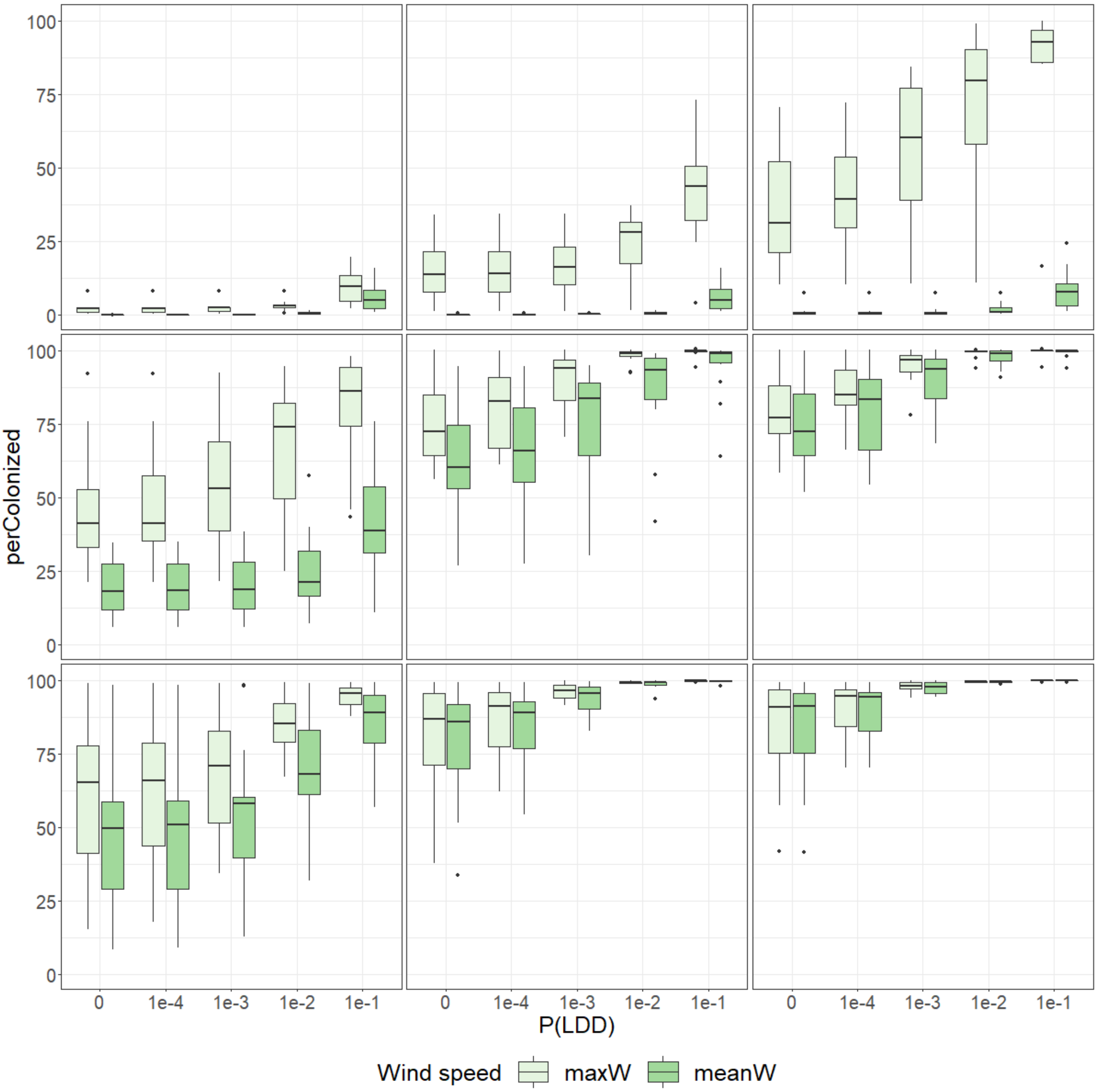

Supplementary Figure 6. Box-plots (showing the 1st and 3^{d} quartiles (upper and lower bounds), 2nd quartile (centre), 1.5* interquartile range (whiskers) and minima-maxima beyond the whiskers) of simulated colonization rates expressed as the ratio (*100), averaged over 30 replicates, between the number of effective colonization events (including effective colonization events that eventually got extinct at the end of the simulation) and the total number of pixels becoming suitable by 2050 in 40 selected bryophyte species in Europe as a function of spore size (a: <20µm; b: 20-50 µm; c: >50 µm), release height Z0, wind speed, and probability of long-distance dispersal P(LDD), with the HadGem2-ES Global Circulation Model under climate change scenario RCP8.5.
