# Supplementary material for: Bryophytes are predicted to lag behind future climate change despite their high dispersal capacities
Source: Nat Commun. 2020 Nov 5;11:5601. doi: 10.1038/s41467-020-19410-8 (PMC7645420; doi:10.1038/s41467-020-19410-8)
Supplement: Supplementary file 3 — Reporting Summary [file 41467_2020_19410_MOESM3_ESM.pdf]

natureresearch

Corresponding author(s): Alain Vanderpoorten

Last updated by author(s): Oct 1, 2020

Reporting Summary

Nature Research wishes to improve the reproducibility of the work that we publish. This form provides structure for consistency and transparency in reporting. For further information on Nature Research policies, see Authors & Referees and the Editorial Policy Checklist .

Statistics

For all statistical analyses, confirm that the following items are present in the figure legend, table legend, main text, or Methods section.

n/a	Confirmed	
☐	☒	The exact sample size (*n*) for each experimental group/condition, given as a discrete number and unit of measurement
☐	☒	A statement on whether measurements were taken from distinct samples or whether the same sample was measured repeatedly
☐	☒	The statistical test(s) used AND whether they are one- or two-sided<br>*Only common tests should be described solely by name; describe more complex techniques in the Methods section.*
☐	☒	A description of all covariates tested
☐	☒	A description of any assumptions or corrections, such as tests of normality and adjustment for multiple comparisons
☐	☒	A full description of the statistical parameters including central tendency (e.g. means) or other basic estimates (e.g. regression coefficient) AND variation (e.g. standard deviation) or associated estimates of uncertainty (e.g. confidence intervals)
☐	☒	For null hypothesis testing, the test statistic (e.g. *F*, *t*, *r*) with confidence intervals, effect sizes, degrees of freedom and *P* value noted<br>*Give P values as exact values whenever suitable.*
☒	☐	For Bayesian analysis, information on the choice of priors and Markov chain Monte Carlo settings
☒	☐	For hierarchical and complex designs, identification of the appropriate level for tests and full reporting of outcomes
☒	☐	Estimates of effect sizes (e.g. Cohen's *d*, Pearson's *r*), indicating how they were calculated

Our web collection on statistics for biologists contains articles on many of the points above.

Software and code

Policy information about availability of computer code

Data collection	No use of software for data collection
Data analysis	R 5.3.5 (R Core Team 2020. R: A language and environment for statistical computing. R Foundation for Statistical Computing, Vienna, Austria. https://www.R-project.org/.).<br>Biomod2 3.3-7 (Thuiller et al. 2016: Ensemble Platform for Species Distribution Modeling. R package version 3.3-7).<br>Ecospat 3.1 (Di Cola et al. 2017. Ecography 40, 774-787).<br>MigClim 1.7 (Engler & Guisan 2009 Divers. Distrib. 15, 590–601 ; Engler et al. 2012. Ecography 35, 872-878). We developed a modified version that implements pixel-specific dispersal kernels depending on local wind conditions and allows for variation in wind conditions through time . Migclim 1.7 is available on GitHub (https://github.com/FlavienCollart/BryophyteDispersion).

For manuscripts utilizing custom algorithms or software that are central to the research but not yet described in published literature, software must be made available to editors/reviewers. We strongly encourage code deposition in a community repository (e.g. GitHub). See the Nature Research guidelines for submitting code & software for further information.

Data

Policy information about availability of data

All manuscripts must include a data availability statement. This statement should provide the following information, where applicable:

- Accession codes, unique identifiers, or web links for publicly available datasets
- A list of figures that have associated raw data
- A description of any restrictions on data availability

Occurrence data are available from Figshare (DOI: 10.6084/m9.figshare.8289650)

Field-specific reporting

Please select the one below that is the best fit for your research. If you are not sure, read the appropriate sections before making your selection.

☐ Life sciences ☐ Behavioural & social sciences ☒ Ecological, evolutionary & environmental sciences

For a reference copy of the document with all sections, see nature.com/documents/nr-reporting-summary-flat.pdf

Ecological, evolutionary & environmental sciences study design

All studies must disclose on these points even when the disclosure is negative.

Study description	The primary aim of this study was to determine whether apparently very efficient dispersers such as bryophytes can track shifts in areas of suitable climate. Using a novel hybrid statistical-mechanistic approach accounting for intraspecific spatial variations in wind conditions and dispersal parameters, we simulated future migrations in Europe under changing climate conditions. For that purpose, a grid of pixel-specific environmental conditions and dispersal kernels, combining information on species dispersal traits, local wind conditions, as well as landscape features affecting dispersal by wind, is generated and used as input in simulations of species dispersal in the landscape under changing climate conditions. More specifically, species distribution data are combined with climatic variables to produce species distribution models that are calibrated under present and projected under future climatic conditions and used to build mechanistic dispersal models. The latter combine species intrinsic features (spore settling velocity and release height) and extrinsic environmental features (mean horizontal wind speed and canopy height) to generate maps of spatially explicit dispersal kernels. Habitat suitability and dispersal kernel maps, updated at regular intervals, are finally combined to parameterize simulations of dynamic range shifts under changing climatic conditions.
Research sample	The European bryophyte flora
Sampling strategy	Because information on bryophyte species distribution is scarce and very heterogeneous, challenging the application of species distribution models, we selected 10 species based upon their representativeness for each of the four main biogeographic elements (i.e., groups of species sharing similar distribution patterns), namely the Arctic-Alpine, Atlantic, Mediterranean, and wide-temperate elements.
Data collection	Distribution data were collected across the entire European range of each species from the Global Biodiversity Information Facility (GBIF) and a thorough review of more than 600 literature sources.
Timing and spatial scale	From 1960 to 2017. We excluded data collected before 1960 for two reasons. First, old records often lack sufficiently precise location information. Second, we wanted to avoid a potential mismatch between old observations and current climate conditions used for modelling. Spatial scale: Europe, on which we focused because of the availability of extensive distribution data.
Data exclusions	We excluded all data without the sufficient level of geolocation precision (arcminute resolution) and restricted our sampling to data collected after 1960 to avoid a potential mismatch between old observations and current climate conditions used for modeling. Only points that were separated by at least 0.1 degree from each other were retained to avoid sampling bias and reduce the risk of spatial autocorrelation.
Reproducibility	To build the Species Distribution Models, 10 replicates were run. For each run, 80 % of the data was used to calibrate the models and the remaining 20 % was kept aside to evaluate the performance of the model using True Skill Statistics (TSS) and Area Under The Curve (AUC) of a ROC plot (Receiver Operating Characteristics). The climatic suitability models exhibited high average TSS and AUC statistics of 0.78 ± 0.12 and 0.93 ± 0.05 respectively, when models were evaluated against test sets corresponding to 20% of the data (cross-validation). These models did not show any apparent signature of overfitting, as only a very slight increase in AUC and TSS (0.81±0.13 and 0.94±0.05, respectively) was observed when these statistics were computed at the level of the entire dataset.
Randomization	To model species distributions, 80% of the species occurrence data (available for each species from Figshare (DOI: 10.6084/m9.figshare.8289650)) was randomly sampled to generate the models (the remaining 20% served to evaluate the models). This procedure was repeated across 10 replicates.
Blinding	Not applicable

Did the study involve field work? ☐ Yes ☒ No

Reporting for specific materials, systems and methods

We require information from authors about some types of materials, experimental systems and methods used in many studies. Here, indicate whether each material, system or method listed is relevant to your study. If you are not sure if a list item applies to your research, read the appropriate section before selecting a response.

Materials & experimental systems

n/a	Involved in the study
☒	☐ Antibodies
☒	☐ Eukaryotic cell lines
☒	☐ Palaeontology
☒	☐ Animals and other organisms
☒	☐ Human research participants
☒	☐ Clinical data

Methods

n/a	Involved in the study
☒	☐ ChIP-seq
☒	☐ Flow cytometry
☒	☐ MRI-based neuroimaging
